# Smoking has disruptive effects on the small bowel luminal microbiome

**DOI:** 10.1038/s41598-022-10132-z

**Published:** 2022-04-14

**Authors:** Gabriela Leite, Gillian M. Barlow, Ava Hosseini, Gonzalo Parodi, Maya L. Pimentel, Jiajing Wang, Alyson Fiorentino, Ali Rezaie, Mark Pimentel, Ruchi Mathur

**Affiliations:** 1 Medically Associated Science and Technology (MAST) Program, Cedars-Sinai, Los Angeles, CA USA; 2 Karsh Division of Gastroenterology and Hepatology, Department of Medicine, Cedars-Sinai, Los Angeles, CA USA; 3 Division of Endocrinology, Diabetes, and Metabolism, Department of Medicine, Cedars-Sinai, Los Angeles, CA USA

**Keywords:** Microbial communities, Gastroenterology, Gastrointestinal system

## Abstract

Tobacco use is the leading preventable cause of cancer, and affects the respiratory, oral, fecal, and duodenal mucosa-associated microbiota. However, the effects of smoking on the duodenal luminal microbiome have not been studied directly. We aimed to compare the duodenal luminal microbiome in never-smokers, current smokers, and ex-smokers who quit ≥ 10 years ago. In a cross-sectional study, current smokers (CS, n = 24) were identified and matched to never-smokers (NS, n = 27) and ex-smokers (XS, n = 27) by age (± 5 years), body mass index (BMI, ± 3 kg/m^2^), and sex. Current antibiotic users were excluded. The duodenal luminal microbiome was analysed in 1 aspirate sample per subject by 16S rRNA gene sequencing. Relative abundances (RA) of families associated with increased duodenal microbial diversity, Prevotellaceae, Neisseriaceae, and Porphyromonadaceae, were significantly lower in CS vs. NS. This was driven by lower RA of unknown *Prevotella* and *Porphyromonas* species, and *Neisseria subflava* and *N. cinerea*, in CS. In contrast, RA of Enterobacteriaceae and Lactobacillaceae (associated with decreased diversity), were significantly higher in CS, due to higher RA of *Escherichia-Shigella*, *Klebsiella* and *Lactobacillus* species. Many of these changes were absent or less pronounced in XS, who exhibited a duodenal luminal microbiome more similar to NS. RA of taxa previously found to be increased in the oral and respiratory microbiota of smokers were also higher in the duodenal luminal microbiome, including *Bulledia extructa* and an unknown *Filifactor* species. In conclusion, smoking is associated with an altered duodenal luminal microbiome. However, ex-smokers have a duodenal luminal microbiome that is similar to never-smokers.

## Introduction

Tobacco use is the leading preventable cause of cancer and cancer deaths^1–3^. Despite increased awareness of these facts, smoking still causes 480,000 deaths per year in the United States alone^2^. Cancers of the lung, in particular non-small cell cancer, and of the mouth, larynx, esophagus, stomach, kidney, pancreas, liver, bladder, cervix, colon and rectum, and acute myeloid leukemia are associated with tobacco smoking^2,4^. An increased prevalence of emphysema, chronic bronchitis, asthma and other pulmonary specific diseases is also seen in long-term smokers^5–7^. Tobacco smoking is also linked to non-pulmonary conditions such as cardiovascular disease^8,9^, cerebrovascular disease^10^, and diabetes^11^, as well as reproductive issues^12^. Smoking also exerts a detrimental effect on the gastrointestinal tract, impacting the mucosal immune response and increasing the risk for Crohn’s disease (CD)^13^.

Given the relationship between smoking and the respiratory system, it is not surprising that smoking has been shown to affect the respiratory tract microbiota. Although human data are sparse, smoking has been suggested to increase the relative abundance of the genera *Veillonella* and *Megasphaera* in the upper respiratory tract microbiome^14^. Clinical studies of respiratory tract microbiota in smoking-related conditions such as lung cancer have shown variable results, likely due to significant differences in the microbiota of the upper and lower respiratory tract^15^, as well as differences in sampling and sequencing techniques among studies. However, a recent meta-analysis suggests increased abundances of the phyla Actinobacteria and Firmicutes in the respiratory microbiota of lung cancer patients, including lower relative abundance of the genus *Prevotella*, and higher *Streptococcus*, when compared to healthy controls^16^. Studies in mice have also shown that exposure to cigarette smoke significantly alters murine respiratory microbiota, with decreases in the genera *Lactobacillus*, *Kluyvera,* and *Nesterenkonia* and increases in *Trichococcus* and *Escherichia-Shigella*^17,18^.

Human studies using stool samples have shown that smoking also affects the fecal microbiome, although the results of these studies are variable. For example, Lee et al. found decreased relative abundance of the phyla Firmicutes and Proteobacteria, and increased relative abundance of Bacteroidetes, in the fecal microbiome of smokers^19^. Further, these changes may be significantly mitigated following smoking cessation^20^.

Within the gastrointestinal tract, the small intestine is primarily responsible for digestion and nutrient absorption, and plays important roles in immune regulation and maintaining gut barrier integrity^21–23^. We have previously shown that stool is a poor surrogate for the small bowel microbiome^24^. However, the small intestine can only be accessed through invasive and expensive techniques such as endoscopy. As a result, few studies of the small intestinal microbiome have been performed to date, and only one explored the impact of smoking on the small intestine^25^. Shanahan et al. examined the effects of smoking on the duodenal mucosa-associated microbiome using duodenal biopsies ^25^, and found that current smokers exhibited significantly decreased bacterial diversity and increased relative abundance of phylum Firmicutes, including species from the genera *Streptococcus* and *Veillonella*, as well as increased relative abundance of the genus *Rothia*, and decreased relative abundance of the genera *Prevotella* and *Neisseria*^25^.

It is known that gut mucosa-associated and luminal microbial populations differ from each other, and fulfill different roles^26–28^. For example, the mucosal microbiota are in close contact with gut epithelial cells, lending to roles in nutrient uptake and immune function, whereas luminal microbes are more involved in digestion^29,30^. In addition, the mucous in the luminal microbiome harbours Gram negative bacteria, including Gram negative species from phylum Proteobacteria which we have consistently found to be disruptors of the small intestinal luminal microbiome^31–33^, but the viscosity of this mucus has been a barrier to fully characterizing luminal microbial populations. We recently developed and validated techniques to optimize DNA yields and 16S rRNA gene sequencing for small intestinal luminal aspirates that break down this mucus, rendering its microbial content more accessible^34^, and have used these in a series of studies to characterize changes in the small intestinal microbiome in small intestinal bacterial overgrowth (SIBO)^31^, with age and the aging process^32^, and with proton pump inhibitor (PPI) use^35^, amongst others.

In this study, we used these validated techniques to characterize and compare the composition of the duodenal luminal microbiome in never-smokers, current smokers, and ex-smokers who had not smoked in 10 or more years.

## Results

### Demographics

Duodenal aspirates from a total of 78 REIMAGINE subjects were included in this study. These included current smokers (CS, N = 24), ex-smokers who had not smoked in 10 or more years (XS, N = 27), and never-smokers (NS, N = 27). Groups were matched by sex (number of males/females), age (± 5 years), and body mass index (BMI, ± 3) (Table 1). Current smokers who provided data reported an average of 17.59 ± 17.44 pack-years of smoking, and ex-smokers who provided data reported an average of 11.16 ± 21.43 pack-years of smoking (see Table 1). The average number of years since quitting for ex-smokers was 26 ± 11.48 years. No subjects were taking antibiotics at the time of endoscopy. Proton pump inhibitor (PPI) use was significantly higher in the CS and XS groups when compared to NS (P = 0.03, Table 1). When the primary reasons for endoscopy were compared between groups, biliary disease, a category which includes pancreatitis, was listed for 7 subjects in the CS group, and further exploration of their charts revealed that these subjects had acute pancreatitis, recurrent pancreatitis, or a history of pancreatitis. These findings indicated an increased prevalence of pancreatitis when compared to never-smokers (P = 0.02, OR: 10.94, 95% Cl: 1.56–127.20). In contrast, the prevalence of pancreatitis was not significantly increased in ex-smokers who had quit smoking for 10 or more years when compared to never-smokers (P = 0.35, OR: 3.41, 95%Cl: 0.47–45.79) (Table 1). None of the other reasons for endoscopy were significantly different among groups.

**Table 1:** Subject characteristics.

Group	Never-smokers (NS)	Current smokers (CS)	Ex-smokers (XS)^1^
N (Males/Females)	27 (18/9)	24 (16/8)	27 (18/9)
Average age, years (range)	52 (24–78)	52 (21–79)	56 (31–69)
Average BMI, kg/m^2^ (range)	24.91 (15.17–35.36)	25.78 (16.73–44.72)	24.45 (17.64–32.92)
Smoking history, average pack-years (range)	0.00	17.59 (0.19–60.00)	11.16 (0.03–92.86)
Years since quitting, average (range)	N/A	N/A	26.00 (10.00–46.00)
Proton pump inhibitor (PPI) use, N (%)	**8 (30%)**	15 (63%)*	15 (56%)**
**Reason for endoscopy, N (%)**^2^			
1. GERD/dyspepsia workup	6 (23%)	5 (22%)	8 (32%)
2. Possible bleeding/anemia workup	0 (0%)	1 (4%)	1 (4%)
3. Rule out cancer/polyps	6 (23%)	5 (22%)	6 (24%)
4. Biliary disease (includes pancreatitis)	**1 (4%)**	7 (30%)*	3 (12%)
5. Dysphagia	6 (23%)	2 (9%)	2 (8%)
6. Crohn’s disease	3 (12%)	0 (0%)	1 (4%)
7. Functional GI disease	2 (8%)	0 (0%)	2 (8%)
8. Rule out Celiac disease	2 (8%)	0 (0%)	0 (0%)
9. Known peptic ulcer disease	0 (0%)	1 (4%)	1 (4%)
10. G-tube management	0 (0%)	1 (4%)	0 (0%)
11. Other	0 (0%)	1 (4%)	2 (8%)

^1^Ex-smokers had not smoked in the last 10 years. ^2^Data available for 26 NS, 23 CS, and 25 XS subjects. Significant differences are shown in bold. * denotes significant differences between CS and NS, and ** denotes significant differences between XS and NS.

### Differences in the duodenal luminal microbiome in current smokers compared to never-smokers

**Figure 1:**
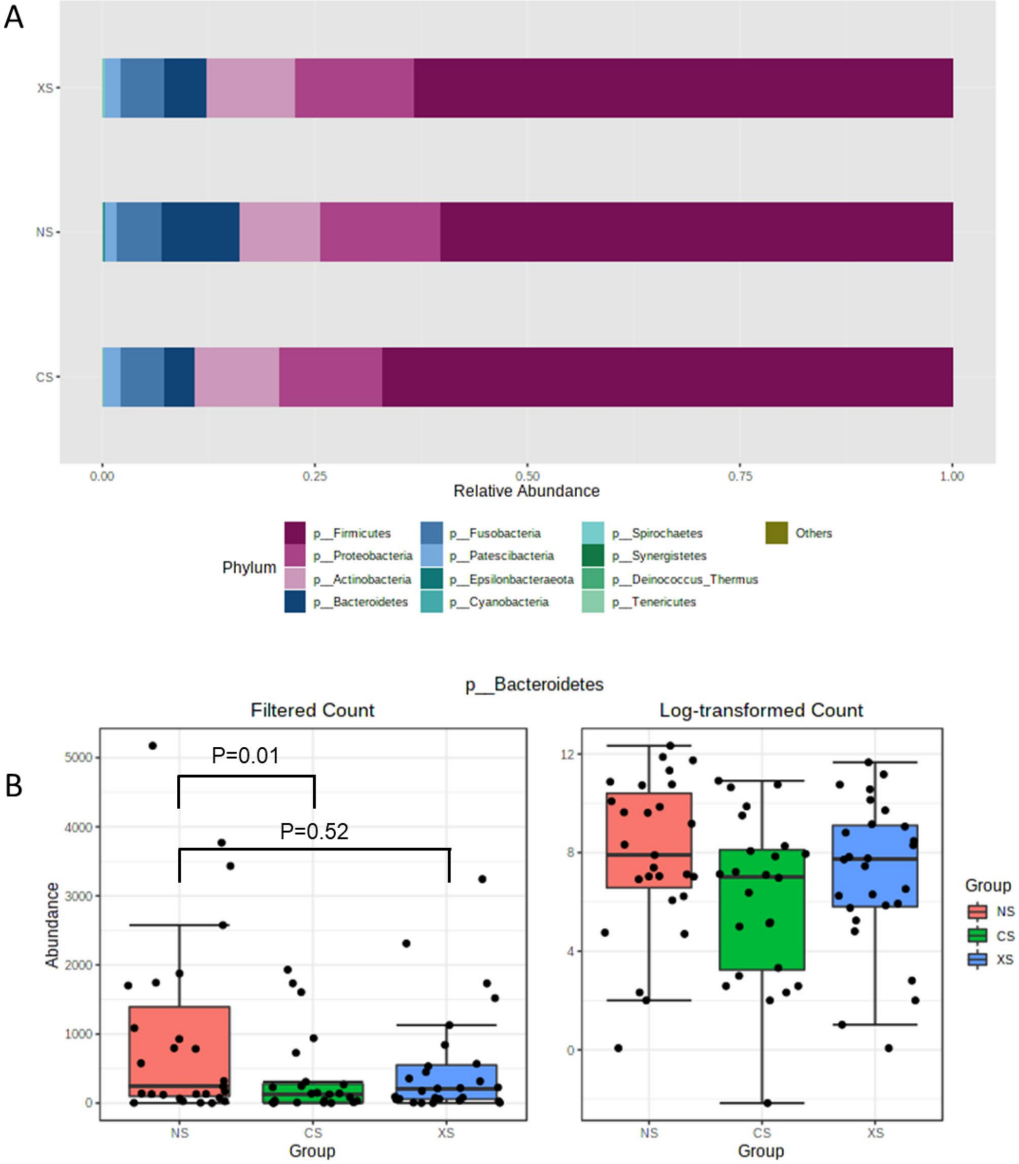
(**A**) Relative abundance of different phyla in the duodenal luminal microbiome of never-smokers (NS), current smokers (CS), and ex-smokers (XS). (**B**) Log-transformed abundance of phylum Bacteroidetes in the duodenal luminal microbiome of NS (red), CS (green), and XS (blue).

The average retained and identified reads with 97% similarity considering the SILVA database (after quality filtering and removal of chimeric reads) did not differ between groups (NS = 199,134; CS = 169,834 and XS = 180,206; P = 0.58). While there were no significant differences in overall microbial alpha and beta diversity in the duodenal luminal microbiome of the CS group when compared to the NS and XS groups (Supplemental Fig. 1A, B), specific taxonomic differences were identified between the groups. At the phylum level, the duodenal luminal microbiome in aspirate samples from all three groups (NS, CS, and XS) was dominated by Firmicutes (~ 63%), followed by the phyla Proteobacteria (~ 13%), Actinobacteria (10%), Bacteroidetes (~ 6%), Fusobacteria (~ 5%), and Patescibacteria (~ 1%) (Fig. 1A). The relative abundance of phylum Bacteroidetes was lower in the CS group (~ 3%) than in the NS group (~ 9%) (fold change (FC) = −3.78, P = 2.2E-3, false discovery rate-adjusted (FDR-adj.) P  = 0.01, Fig. 1B). Although the same trend was observed when the XS group was compared to the NS group (FC = −1.68, P = 0.22, FDR-adj. P  = 0.52), the relative abundance of Bacteroidetes in the duodenal luminal microbiome of the XS group (~ 5%) was closer to that in the NS group (~ 9%, Fig. 1B).

**Figure 2:**
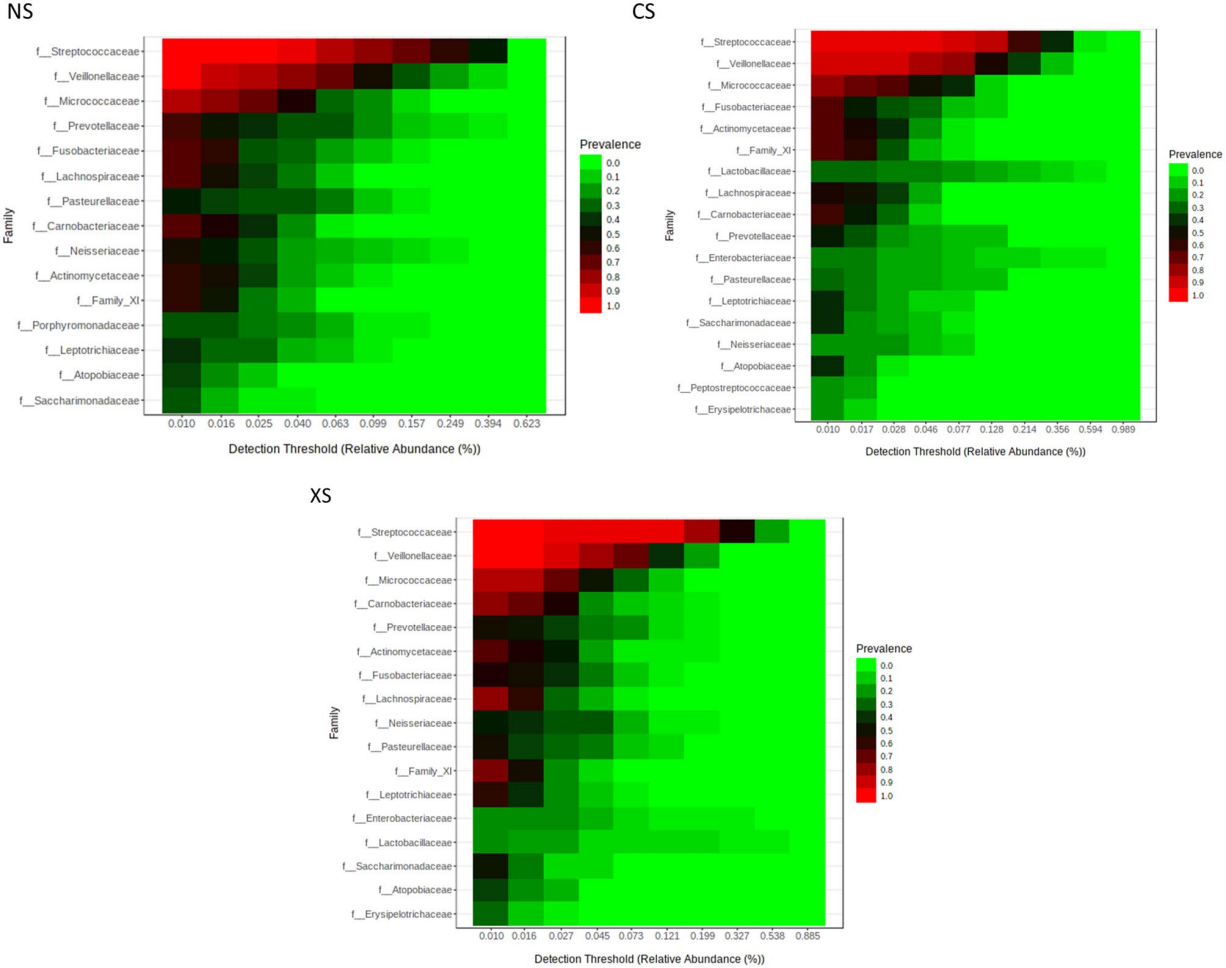
The common duodenal luminal microbiome at the family level in never-smokers (**A**), current smokers (**B**) and ex-smokers (**C**).

**Figure 3:**
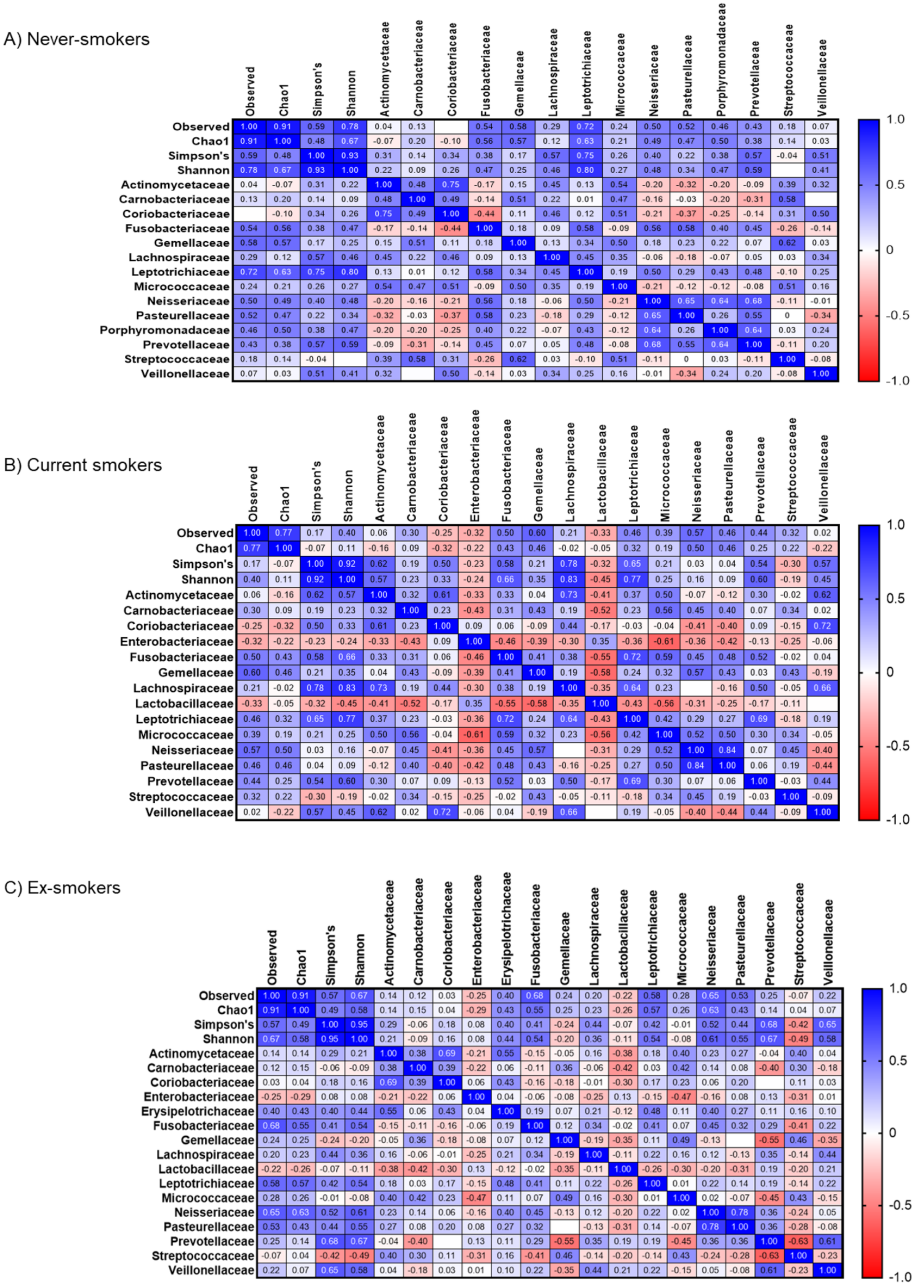
Spearman associations between duodenal microbial families in never-smokers (**A**), current smokers (**B**), and ex-smokers (**C**).

**Figure 4:**
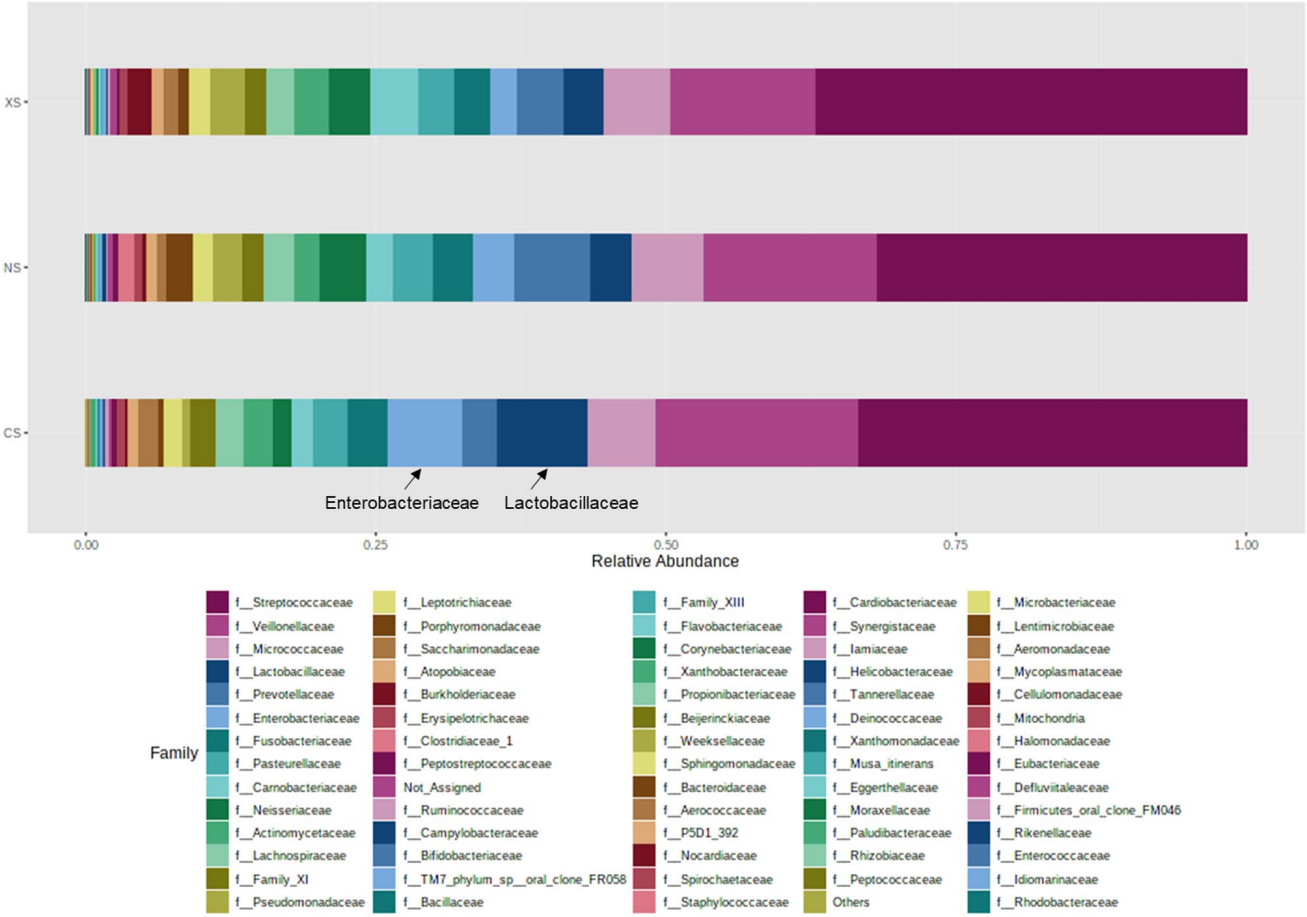
Relative abundance of different families in the duodenal luminal microbiome of never-smokers (NS), current smokers (CS), and ex-smokers (XS). Families Enterobacteriaceae and Lactobacillaceae are highlighted.

The common duodenal luminal microbiome was defined as the most widespread components of the microbiome found across at least 40% of subjects. At the family level, this common duodenal luminal microbiome was significantly different in the CS group when compared to the NS group (Fig. 2A,B). The prevalences of certain duodenal families were found to be positively associated with microbial diversity, including Prevotellaceae (phylum Bacteroidetes), Neisseriaceae (phylum Proteobacteria) and Porphyromonadaceae (phylum Bacteroidetes) (Fig. 3A), and the relative abundances of these families were lower in the CS group than in the NS group (Prevotellaceae FC = −2.80, P = 0.03, FDR-adj. P = 0.05, Neisseriaceae FC = −3.41, P = 9.45E-3, FDR-adj. P = 0.05, and Porphyromonadaceae FC = −6.53, P = 5.36E-4, FDR-adj. P = 4.37E-3) (Fig. 4, Supplemental Table 1). As a result, Porphyromonadaceae was no longer part of the common duodenal luminal microbiome in the CS group (Fig. 2B). The prevalences of other duodenal microbial families were found to be associated with decreased microbial diversity, including Enterobacteriaceae (phylum Proteobacteria) and Lactobacillaceae (phylum Firmicutes) (Fig. 3B). The relative abundance of family Enterobacteriaceae was higher in the duodenal microbiome of the CS group than in the NS group (Enterobacteriaceae FC = 14.77, P = 1.32E-4, FDR-adj. P = 1.37E-3, Supplemental Table 1). The relative abundance of family Lactobacillaceae also trended towards being higher in the CS group vs. the NS group, but the P-value reached significance only when a non-parametric statistical test was used (FC = 2.08, Mann–Whitney P = 0.036) (Fig. 4, Supplemental Table 1).

Analysis of Spearman correlations suggested that the families Enterobacteriaceae and Lactobacillaceae had negative associations with most families in the common duodenal luminal microbiome of the CS group, including Porphyromonadaceae, Prevotellaceae and Neisseriaceae (Fig. 3B, Supplemental Fig. 2), and that there were significantly more negative associations among families overall in the CS group than in the NS group (Supplemental Fig. 2).

### Differences in the duodenal luminal microbiome are reduced in ex-smokers

Many of the differences seen in the duodenal luminal microbiome of the CS group when compared to the NS group were not seen or were less pronounced in the XS group. As a result, the duodenal luminal microbiome profile in the XS group was more similar to that of the NS group (Fig. 2C, Supplemental Table 1). This included similar relative abundances of the disruptors Enterobacteriaceae and Lactobacillaceae (Supplemental Fig. 3) and of families associated with duodenal microbial diversity (Porphyromonadaceae, Prevotellaceae and Neisseriaceae). Analysis of Spearman correlations suggested a higher number of positive associations between families in the XS group (Fig. 3C, Supplemental Fig. 3). Of note, the XS group in this study was limited to subjects who had quit smoking 10 or more years ago.

### Differences are seen in duodenal luminal genera and species in current smokers, never-smokers, and ex-smokers

Analyses at the genus and species levels revealed that the higher relative abundance of family Enterobacteriaceae in the duodenal luminal microbiome of the CS group appeared to be primarily driven by the Gram-negative genera *Escherichia-Shigella* and *Klebsiella.* The relative abundance of an unknown species from genus *Escherichia-Shigella* was 20.51-fold higher in the duodenal microbiome of the CS group compared to the NS group (FDR-adj. P = 1.20E-3), but was lower in the XS group compared to CS (FC = −7.68, FDR-adj. P = 1.06E-9) (Supplemental Table 1). As a result, the relative abundance of this unknown *Escherichia-Shigella* species was even lower in the XS group than in the NS group (FC = −3.33, FDR-adj. P = 0.02). The relative abundance of the unknown species from genus *Klebsiella* was significantly higher in the CS group when compared to the NS group (FC = 9.9, FDR-adj. P = 0.02, Supplemental Table 1), and was lower in the XS group compared to the CS group (FC = −3.40, FDR-adj. P = 0.02). There was no significant difference in the relative abundance of this species in the XS group when compared to the NS group (P = 0.1, Supplemental Table 1).

**Figure 5:**
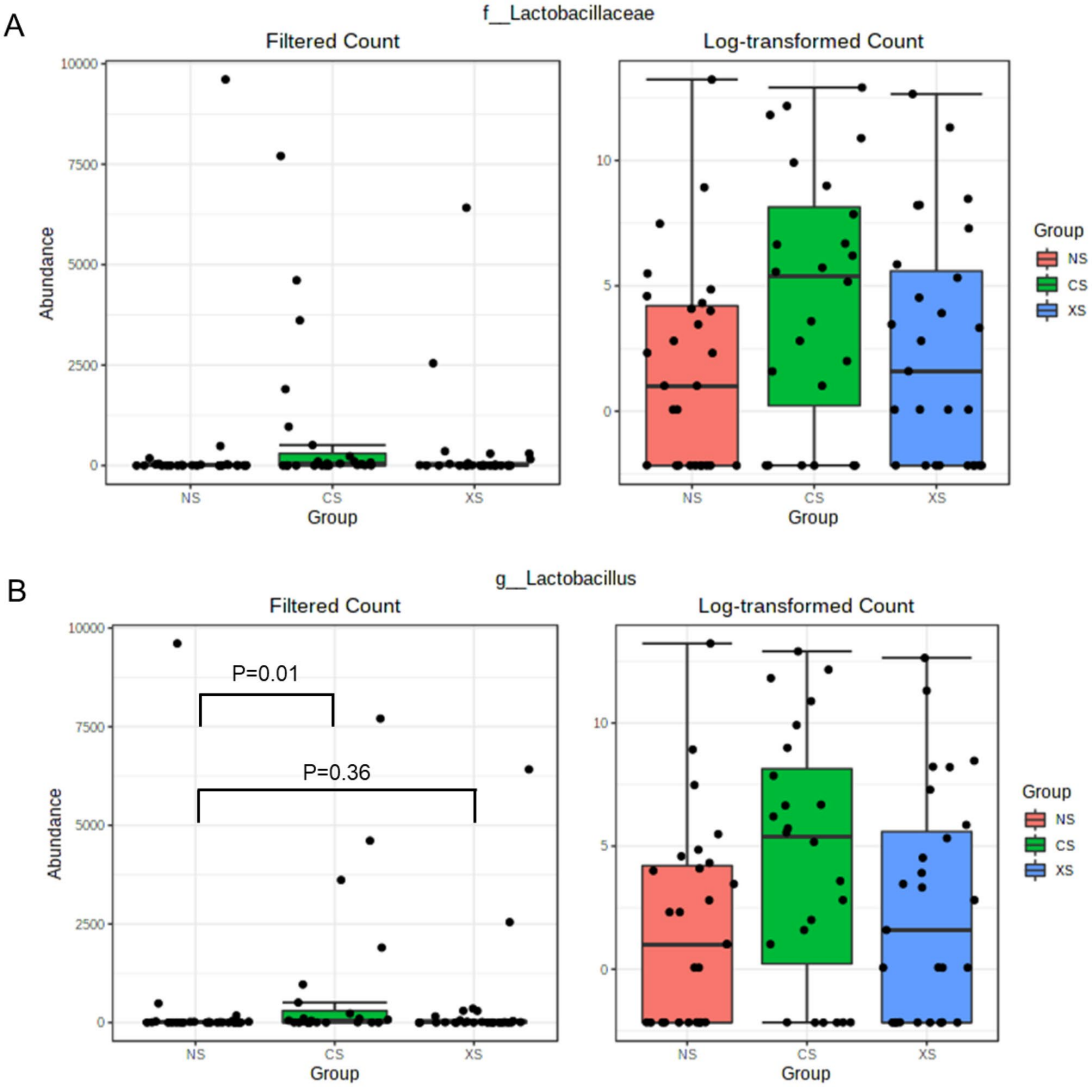
Log-transformed abundances of family Lactobacillaceae (**A**) and genus *Lactobacillus* (**B**) in the duodenal luminal microbiome of never-smokers (NS) (red), current smokers (CS) (green), and ex-smokers (XS) (blue).

The higher relative abundance of family Lactobacillaceae (phylum Firmicutes) in the CS group was driven by several species from the genus *Lactobacillus*, which was 4.81-fold higher in the CS group when compared to the NS group (Fig. 5A, FDR-adj. P = 0.01, Supplemental Table 1). The top 3 species identified as most likely to be *Lactobacillus* that had higher relative abundance in the CS group when compared to NS group (of a total of 11) were *L. panis* (FC = 6.4, FDR-adj. P = 2.65E-7), *L. oris* (FC = 6.88, FDR-adj. P = 4.62E-7) and *L. murinus* (FC = 7.28, FDR-adj. P = 2.38E-6). In addition, the relative abundance of genus *Lactobacillus* tended to be higher in the CS group when compared to the XS group, but this did not reach statistical significance (FC = 2.17, P = 0.06, FDR-adj. P = 0.28, Fig. 5A, Supplemental Table 1). There was no significant difference in the relative abundance of genus *Lactobacillus* in the XS group when compared to the NS group (Fig. 5B, P = 0.1, FDR-adj. P = 0.36).

Changes in family Prevotellaceae were partially due to an unknown species from genus *Prevotella*, the relative abundance of which was significantly lower in the CS group relative to the NS group (FC = −19.6, FDR-adj. P = 9.84E-7), but interestingly, was higher in the XS group when compared to the CS group (FC = 10.81, FDR-adj. P = 7.15E-3, Supplemental Fig. 4, Supplemental Table 1). The relative abundance of this unknown *Prevotella* species in the XS group was closer to that in the NS group (FC = −1.81, FDR-adj. P = 0.02, Supplemental Fig. 4, Supplemental Table 1).

The lower relative abundance of family Neisseriaceae in the duodenal luminal microbiome in the CS group vs. the NS group was partially due to two commensal species, *Neisseria subflava* (FC = −4.17, FDR-adj. P = 8.36E-4) and *N. cinerea* (FC = −5.46, FDR-adj. P = 8.50E-4) (Supplemental Table 1). The relative abundances of these species were not significantly different in the XS group when compared to the NS group (*N. cinerea* FC = −1.33, P = 0.11, FDR-adj. P = 0.44; *N. subflava* FC = −1.66, P = 0.07, FDR-adj. P = 0.34) (Supplemental Fig. 5, Supplemental Table 1). Of note, Neisseria species were classified using Greengenes Database 2013 because these species could not be identified using the SILVA Database v132.

The relative abundance of family Porphyromonadaceae, which was part of the core duodenal luminal microbiome in the NS group, fell out of the core duodenal microbiome in the CS group (Fig. 2B) and in the XS group (Fig. 2C). This was partially due to lower relative abundance of an unknown species from genus *Porphyromonas*. The relative abundance of this unknown *Porphyromonas* species was lower in the CS group relative to the NS group (FC = −3.86, FDR-adj. P = 9.15E-5), and was also lower in the XS group when compared to the NS group (FC = −2.03, P = 0.01, FDR-adj. P = 0.08).

In addition to *Porphyromonas* species, the relative abundances of other taxa from phylum Bacteroidetes were lower in the CS group relative to the NS group, and were also lower in the XS group, 10 or more years after quitting smoking. These included genus *Chryseobacterium* (family Weeksellaceae), which was lower in the CS group than in the NS group (FC = −4.06, P = 3.26E-4, FDR-adj. P = 2.64E-3, Supplemental Table 1), but was not significantly different in the XS group when compared to the NS group (P = 0.69, FDR-adj. P = 1, Supplemental Table 1).

Although no statistical differences were observed in Kyoto Encyclopedia of genes and Genomes (KEGG) predicted microbial metabolic pathways when analyses were performed across all three groups and between group pairs (Supplemental Tables 2 to 5), discovery analysis based on microbial pathways enriched in the CS group as compared to the NS group revealed a pattern associated with toluene and xylene degradation (linear discriminant analysis (LDA) scores > 2) (Supplemental Fig. 6).

### The duodenal luminal microbiome of current smokers has a microbial signature similar to the oral and respiratory microbiota reported in smoking-related conditions

As the duodenal luminal microbiome can be colonized by a variety of oral and respiratory microbes, and given the direct relationship between tobacco smoking, the oral and respiratory microbiomes^17,18,36^, and the development of certain human diseases^5–7^, microbial taxa previously found to be associated with smoking-related diseases were also quantitated in the duodenal aspirates from our subjects.

The relative abundance of species *Bulleidia extructa* (phylum Firmicutes), which is five times more prevalent in the subgingival microbiome of subjects with smoking-associated periodontitis than in healthy controls^37^, was significantly higher in the duodenal luminal microbiome of the CS group when compared to the NS group (FC = 4.55, FDR-adj. P = 1.00E-4). While the relative abundance of *B. extructa* was lower in the XS group than in the CS group (FC = −2.47, P = 0.01, FDR-adj. P = 0.09), it was still significantly higher in the XS group than in the NS group (FC = 3.25, FDR-adj. P = 7.74E-3, Supplemental Table 1).

In addition, the relative abundance of an unknown species from genus *Filifactor* (phylum Firmicutes), which is more abundant in the subgingival microbiome of smokers vs. never-smokers in subjects with chronic moderate periodontitis^38^, was also higher in the duodenal luminal microbiome of the CS group compared to the NS group (FC = 2.21, P = 0.04, FDR-adj. P = 0.2), and lower in the XS group when compared to the CS group (FC = −5.88, FDR-adj. P = 2.03E-6, Supplemental Table 1). The relative abundance of this species was also lower in the XS group when compared to the NS group (FC = −3.67, FDR-adj. P = 4.87E-3, Supplemental Table 1).

## Discussion

In this study, we demonstrate that the duodenal luminal microbiome in current smokers is significantly different from that in never-smokers. Given the critical roles played by small intestinal microbes in digestion, nutrient absorption, immune regulation, and maintenance of gut barrier integrity^23,39^, coupled with the fact that an estimated 942 million men and 175 million women ages 15 or older are current smokers^40^, these findings suggest that smoking may have implications for metabolic and gastrointestinal health beyond the conditions currently recognized. Interestingly, our findings also indicate that many, but not all, of the alterations in the duodenal luminal microbiome in current smokers were less pronounced or absent in ex-smokers who had stopped smoking 10 or more years earlier, which may suggest that the effects of smoking are lessened after 10 or more years of stopping smoking.

Although there were no phylum-level changes that reached significance between the three groups (current smokers (CS), never-smokers (NS), and ex-smokers who had not smoked in 10 or more years (XS)), there was a trend towards lower relative abundance of Bacteroidetes in the CS group when compared to the NS group, and to a lesser extent in the XS group when compared to the NS group. Analysis at the family level led to a number of interesting and statistically significant findings. We found that the core duodenal luminal microbiome, i.e. the most widespread components of the microbiome found in all subjects, was significantly altered in the CS group when compared to the NS group. Specifically, we found that certain bacterial families were positively correlated with microbial diversity, including Porphyromonadaceae (phylum Bacteroidetes), Prevotellaceae (phylum Bacteroidetes), and Neisseriaceae (phylum Proteobacteria), and that the relative abundances of all three of these families were significantly lower in current smokers than in never-smokers. The lower relative abundances of these three families appeared to be driven primarily by higher relative abundances of taxa from two other bacterial families, Lactobacillaceae (phylum Firmicutes) and Enterobacteriaceae (phylum Proteobacteria), and were associated with a greater number of negative associations between microbial families in current smokers. Our studies of the small intestinal luminal microbiota have consistently shown that increased relative abundance of both Enterobacteriaceae and Lactobacillaceae can disrupt microbial diversity and significantly impact microbial profiles ^31–33^, and our current findings further support this.

An encouraging finding was that many of the negative changes in the duodenal luminal microbiome identified in current smokers appear to be significantly less pronounced or absent in ex-smokers who had not smoked in 10 or more years. As a result, the duodenal microbial profile in the XS group was more similar to the NS group than the CS group, with fewer negative associations between families. At the family level, there were no significant differences in the overall relative abundances of Prevotellaceae, Neisseriaceae or Porphyromonadaceae in the XS group when compared to the NS group. The relative abundances of the disruptive genera *Klebsiella* and *Lactobacillus* (most likely *L. panis*, *L. oris*, and *L. murinus*) were higher in the CS group, but were not significantly different in the XS group when compared to the NS group. The relationship between these findings and the documented mitigation of smoking-associated disease risks in ex-smokers^1–3^ remains to be determined.

The relative abundances of unknown species from the genera *Prevotella* and *Porphyromonas* were lower in both the CS and the XS groups than in the NS group, and the relative abundance of *Bulledia extructa* was higher in both the CS and the XS groups than in the NS group, suggesting that these might represent permanent changes in the duodenal microbiome of former smokers. The relative abundance of the disruptive genus *Escherichia-Shigella* was even lower in the XS group than in the NS. We hypothesized that this may represent a reconfiguration of the small intestinal luminal microbiome in ex-smokers who have quit for 10 or more years. As subjects taking antibiotics were excluded from the study, this is not an antibiotic effect, but further explorations are required to determine the reasons for this difference.

It is increasingly understood that there is considerable bi-directional cross-talk between the gut and the lungs, referred to as the ‘gut-lung axis’, and that gut microbiota can influence the lungs and lung immune responses through the effects of microbial components (such as lipopolysaccharides (LPS)) and metabolites (such as short chain fatty acids (SCFA))^41,42^. Supporting this, individuals with gut conditions such as inflammatory bowel disease (IBD) have increased prevalence of pulmonary diseases^43^, and individuals with chronic obstructive pulmonary disease (COPD), cystic fibrosis (CF), and asthma have increased prevalence of gastrointestinal conditions including irritable bowel syndrome (IBS)^44,45^. Interestingly, some of the microbes we found to be altered in the duodenal luminal microbiome of the CS group have previously been shown to be altered in the respiratory microbiota of smokers and individuals with pulmonary conditions^46^. For example, the relative abundance of the genera *Neisseria* and *Porphyromonas* is lower both in the duodenal luminal microbiome of our CS group and in the respiratory microbiome of smokers^46^. Genus *Prevotella*, which is highly abundant in the respiratory microbiome^46^, was lower in the CS group and is also noted to be decreased in the respiratory microbiome of lung cancer patients^16^ and the fecal microbiome of tuberculosis patients^47^. Lastly, we identified higher relative abundance of *Enterobacteriaceae* and an *Escherichia-Shigella* species (likely *E. coli*) in the duodenal luminal microbiome of our CS group, both of which are also higher in the gut microbiota of mice with respiratory influenza infections^48^. Taken together, these results demonstrate that some of the microbial changes we identified in the duodenal luminal microbiome of smokers parallel differences seen in respiratory and fecal microbiota of smokers and individuals with lung cancer and other pulmonary conditions, as well as in animal models, and may warrant further investigation.

There are also consistencies between our findings and changes in the oral microbiota in smokers^36^. We found that the relative abundance of *B. extructa* (phylum Firmicutes) and an unknown species from genus *Filifactor* (phylum Firmicutes) was higher in the CS group, both of which are associated with periodontitis and further increased in the subgingival microbiome of smokers with periodontitis^37,38^. The relative abundance of *Klebsiella* was also higher in the CS group, which is consistent with an early study which suggested that cigarette smoking promoted the growth of Gram-negative bacteria, including *Klebsiella* species, in the oral cavity^49^, and another study which found that *K. pneumoniae* was the second most commonly identified bacterial species in sputum of smokers with lung cancer that develop pneumonia^50^. Lastly, the relative abundance of *Lactobacillus* was higher in the CS group, and is also higher in the oral microbiome of current smokers^36^. Interestingly, changes in the small bowel microbiome in the CS group were linked to changes in predicted microbial pathways associated with toluene and xylene degradation, two volatile organic compounds (VOCs) present in tobacco smoke^51^. These findings may suggest that smoking has effects on the microbiome along an extended length of the gastrointestinal tract.

Like the lungs, the duodenum was thought until recently to have relatively low microbial biomass^52^. In fact, the true biomass is hard to determine as the viscosity of small intestinal samples creates challenges for microbial recovery, especially when using kits designed for stool samples. To combat this, we recently developed and validated novel techniques that facilitate increased recovery of microbes from small intestinal luminal aspirates, as well as improved DNA isolation and library preparation for 16S rRNA sequencing^34^. Through the use of these techniques, we have shown that there are greater numbers of microbes in duodenal luminal fluid than previously thought, and that the microbial populations in duodenal luminal fluid are significantly different from those in stool^24,33^. Moreover, luminal microbial populations also differ in the different segments of the small intestine^24^.

Shanahan et al.^25^ previously examined the effects of smoking on the duodenal mucosa-associated microbiome, and there are similarities and differences between those findings and our findings regarding the duodenal luminal microbiome. Shanahan et al. found significantly lower bacterial diversity in the duodenal mucosa-associated microbiome, as well as higher relative abundance of phylum Firmicutes, and lower relative abundance of Bacteroidetes and Actinobacteria, in current smokers vs. never-smokers^25^, whereas we did not find any significant differences in overall microbial alpha and beta diversity in the duodenal luminal microbiome of current smokers when compared to never-smokers and ex-smokers. We also did not find any significant differences at the phylum level, although there was a trend towards lower relative abundance of phylum Bacteroidetes in the CS group. At the genus level, Shanahan et al.^25^ found significantly higher relative abundance of the genera *Streptococcus*, *Veillonella*, and *Rothia* in duodenal mucosa-associated microbiome of current smokers vs. never-smokers, but we did not find significant differences in these genera in the duodenal luminal microbiome. We found higher relative abundances of *Lactobacillus,* and the Gram-negative genera *Escherichia-Shigella* and *Klebsiella*, in the duodenal luminal microbiome of current smokers, but Shanahan et al. did not report alterations in these genera in the duodenal mucosa-associated microbiome^25^. These differences are not unexpected, given the differences in luminal vs. mucosal microbial populations previously noted^26,27^. Importantly, there were also a number of consistent findings, including the lower relative abundance of *Prevotella* and *Neisseria* in current smokers vs. never-smokers, although the similar relative abundances of *Lactobacillus* and *Klebsiella* species we found in ex-smokers and never-smokers in our cohort were not reported by Shanahan et al.^25^. All of the ex-smokers in our cohort had quit smoking 10 or more years ago, which may explain why these and other taxa we found to be altered in current smokers were more similar in ex-smokers and never-smokers.

Our study has limitations. The subjects were undergoing upper endoscopy for a variety of reasons, including assessment of intestinal complaints as well as for screening purposes due to familial and other risk factors, and may not be fully representative of normal healthy individuals. Sample sizes were also limited, which may have affected some of the post correction tests applied to P-values during metagenomic and pathway prediction analyses, as a large sample size is usually required to achieve good power while controlling for false positives at a low level (i.e. low false discovery rate (FDR))^53^. In addition, this was a cross-sectional study with a single sample per subject. Current smokers were matched to ex-smokers and never-smokers by age, BMI, and gender, antibiotic users were excluded, and other potential influences on the gut microbiome, including PPI use and reasons for endoscopy, were also compared between groups. However, we cannot rule out other potential confounders that could affect the microbiome over time in each of the groups, and larger studies would be required to address this fully. PPI use was significantly higher in the CS and XS groups when compared to the NS group, which is consistent with previous findings of increased prevalence of PPI use amongst smokers^54,55^. However, we did not find any significant differences in those small intestinal luminal taxa we have previously identified as affected by PPI use^35^, suggesting that our current findings are not driven by PPI use. Apart from the higher prevalence of pancreatitis in current smokers, which is consistent with the fact that smoking is a known risk factor for pancreatitis^56^, there were no other significant differences in primary diagnosis/reason for endoscopy between the groups.

In conclusion, this study demonstrates that smoking affects the duodenal luminal microbiome, and many of the differences in the duodenal luminal microbiome in current smokers and never-smokers are not seen, or are significantly less pronounced, in ex-smokers. We found that smoking is associated with significantly higher relative abundances of the disruptive genera *Escherichia-Shigella*, *Klebsiella* and *Lactobacillus*, as well as lower relative abundance of genera associated with microbial diversity such as *Prevotella* and *Neisseria*, both of which are also lower in the duodenal mucosa-associated microbiome^25^*.* In addition, some of the taxa we identified as having higher relative abundance in the duodenal luminal microbiome of smokers are similar to alterations in the oral microbiota of smokers, particularly smokers with periodontitis, as well as alterations in the respiratory and fecal microbiota of smokers, lung cancer patients, and patients with various respiratory conditions, raising the possibility that they may represent perturbations of the gut-lung axis in smokers. While many of the duodenal luminal microbiome differences seen in current smokers were not seen in ex-smokers who had stopped smoking 10 or more years ago, some appeared to be permanent. While these findings may have implications for small intestinal functions in metabolic and gastrointestinal health, the clinical implications of these changes remain to be determined.

## Methods

### Study subjects

Subjects for this study were identified from the REIMAGINE (Revealing the Entire Intestinal Microbiota and its Associations with the Genetic, Immunologic, and Neuroendocrine Ecosystem) study. REIMAGINE is an ongoing study to explore the impact of alterations in the small bowel microbiota on different conditions and diseases. Subjects between the ages of 18–85 years old undergoing a standard of care upper endoscopy (esophagogastroduodenoscopy – EGD) without colon preparation are eligible for inclusion^31,34^. The study protocol is approved by the Institutional Review Board at Cedars-Sinai Medical Center (Pro00035192), and all subjects provide informed written consent prior to participation.

REIMAGINE subjects complete a detailed questionnaire following the EGD. This questionnaire includes self-documented family and medical history, and includes smoking history^24,31,34^. All medical information provided by subjects is verified by previous medical records. Subjects who completed the smoking section of the questionnaire were eligible for inclusion in this study. Based on their responses, current smokers (CS), never-smokers (NS), and ex-smokers (XS) who had not smoked in 10 or more years were identified. The cut-off of 10 or more years for ex-smokers was chosen as the U.S. Department of Health and Human Services suggests that the risks for cancers of the bladder, esophagus, or kidney decrease within 10 years of quitting smoking, and the risk for lung cancer decreases by 50% within 10–15 years of quitting smoking^1^. Groups were matched by age (± 5 years), body mass index (BMI) (± 3 kg/m^2^), and sex. All data were de-identified prior to analysis. Subjects were excluded if current antibiotic use was reported or if subjects had used antibiotics within 6 months prior the procedure.

### Duodenal luminal samples

Luminal fluid was aspirated from the duodenum during the EGD procedure, using a sterile aspiration double-lumen catheter (Hobbs Medical, Inc.) specifically designed for small bowel aspiration^34^. To minimize the chance of contamination from the mouth, esophagus, and stomach, the endoscopist collected duodenal aspirates using a sterile inner catheter that was manipulated through a sterile bone wax cap only after reaching the second portion of the duodenum^34^.

### Aspirate processing

Duodenal aspirates were mixed with an equal volume of sterile 1X dithiothreitol (DTT) and vortexed for about 30 s until liquified as described previously^34^. The samples were then centrifuged at maximum speed (> 13,000 RPM) for 5 min, followed by the removal of the supernatant. 1 mL of sterile All Protect reagent (Qiagen, Hilden, Germany) was added to the microbial pellet. The pellets under All Protect were then stored at −80 °C until the DNA extraction was performed^34^.

### DNA extraction and isolation

The duodenal aspirate pellets were thawed on ice and processed as previously described^34^. Briefly, microbial DNA was isolated from each sample using the MagAttract PowerSoil DNA KF Kit (Qiagen) on a KingFisher Duo (Thermo Fisher Scientific, Waltham, MA, USA)^34^. DNA quantification was carried out using the Qubit™ ds high sensitivity DNA BR Assay kit (Invitrogen by Thermo Fisher Scientific) on a Qubit™ 4 Fluorometer (Invitrogen, Carlsbad, CA, USA).

### Library preparation and 16S rRNA sequencing and analysis

The 16S V3 and V4 regions were amplified with specific primers (S-D-Bact-0341-b-S-17 and S-D-Bact-0785-a-A-21)^57^ modified to include Illumina sequencing adapters^34^. Library quality was determined on an Agilent 2100 Bioanalyzer System. The MiSeq System (Illumina, San Diego, California) was used to perform paired-end sequencing of amplicons (2 × 301 cycles) with the MiSeq reagent v3 Kit with 600 cycles and 5% to 10% PhiX (Illumina). 16S V3 and V4 libraries from blank samples were also sequenced, as previously described^34^.

### Sequencing and statistical analysis

CLC Genomic Workbench v. 10.1.1 and CLC Microbial Genomics Module v. 2.5 (Qiagen) software was used for Operational Taxonomic Unit (OTU) clustering and taxonomic analysis against the SILVA database v132 with 97% similarity. Greengene Database 2013 v.13_8 was used only where species level classifications were not available in the SILVA database. Default parameters were used for minimum occurrences (2) and chimera crossover cost (3), and the creation of new OTUs was not allowed. Low depth samples (< 5,000 sequences per sample) were removed from the analysis. Microbial alpha diversity indices including Shannon’s, Simpson’s, and Observed were calculated after low depth samples were removed from the analysis. Inter-sample variability (beta diversity) was calculated using the Bray–Curtis metric using MicrobiomeAnalyst^58,59^ tools.

Predictions for significant differentially abundant OTUs between different groups were performed using a rarefied OTU table. Multiple comparisons and statistical analyses were performed using CLC Microbial Genomics Module v.2.5 (Qiagen) and MicrobiomeAnalyst^58,59^. The OTU table was rarefied to the minimal number of reads assigned to a sample, and a Negative Binomial GLM model was used to obtain maximum likelihood estimates for the fold change (FC) of a log-transformed OTU between different groups. The Wald test was used for determination of significance, and P-values were corrected using False Discovery Rate (FDR). PERMANOVA analysis with Bonferroni-corrected P-values (permutations: 99,999) was used to compare beta diversity differences between groups. FC were log-transformed. A P-value < 0.05 after controlling for FDR or Bonferroni was considered to be statistically significant. Two-tailed Spearman r correlations, Mann–Whitney tests, and graph construction were carried out with rarefied OTU tables using GraphPad Prism 7.02 (GraphPad Software, La Jolla, CA, USA) and IBM SPSS Statistics Version. Fisher’s Exact test was used to determine associations between categorical variables between different groups using GraphPad Prism 7.02 (GraphPad Software).

Core microbiome (common microbiome) analysis and correlation constructions were performed using the Core Microbiome tool and correlation network tool available at MicrobiomeAnalyst^58,59^. Relative abundances were calculated based of the percentage of a given taxon in the total taxa identified in the duodenal luminal microbiome. The core microbiome at phylum level and family level was calculated based on the most common phyla in at least 40% of samples. Correlations and associations between microbial taxa were generated based on Spearman rank correlation, using a correlation threshold of at least 0.3 and P-values lower than 0.05. Microbial metabolic pathways were analyzed based on Kyoto Encyclopedia of genes and Genomes (KEGG) collection databases, using PICRUSt2^60^. Microbial predicted pathways were compared across all three groups using the Kruskal–Wallis test and between two groups using the Mann–Whitney test. P-values were adjusted using FDR. Association analyses were also performed using the globaltest algorithm^61^ available at MicrobiomeAnalyst. Linear Discriminant Analysis (LDA) Effective Size (LEfSe)^62^ (Galaxy Version 1.0) was used to discover metagenomic biomarkers for predicted pathways.

### Ethics approval

This study was performed in accordance with the principles of the Declaration of Helsinki. The study was approved by the Institutional Review Board at Cedars-Sinai Medical Center (Pro00035192).

### Consent to participate

Informed written consent was obtained from all individual participants included in the study.

## Supplementary Information

Supplementary Information.

## Data Availability

The datasets generated during this study are available at the National Center for Biotechnology Information (NCBI) BioProject Repository (https://www.ncbi.nlm.nih.gov/bioproject) under BioProject ID PRJNA732897.
