# Supplementary material for: Smoking has disruptive effects on the small bowel luminal microbiome
Source: Sci Rep. 2022 Apr 14;12:6231. doi: 10.1038/s41598-022-10132-z (PMC9010470; doi:10.1038/s41598-022-10132-z)
Supplement: Supplementary file 1 — Supplementary Information. [file 41598_2022_10132_MOESM1_ESM.pdf]

Supplemental Information

A

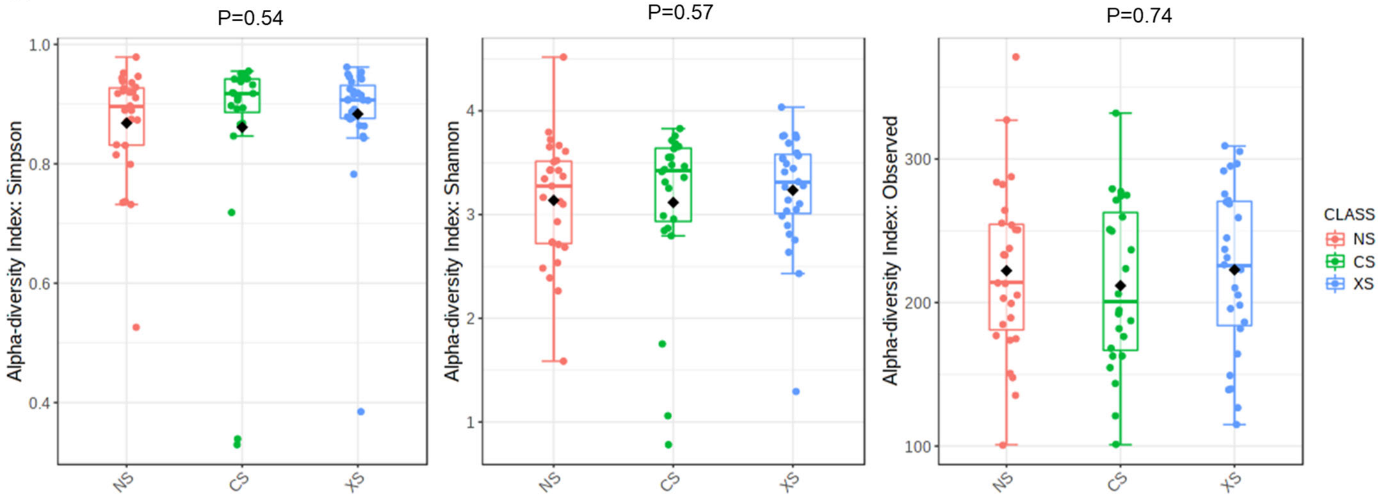

B

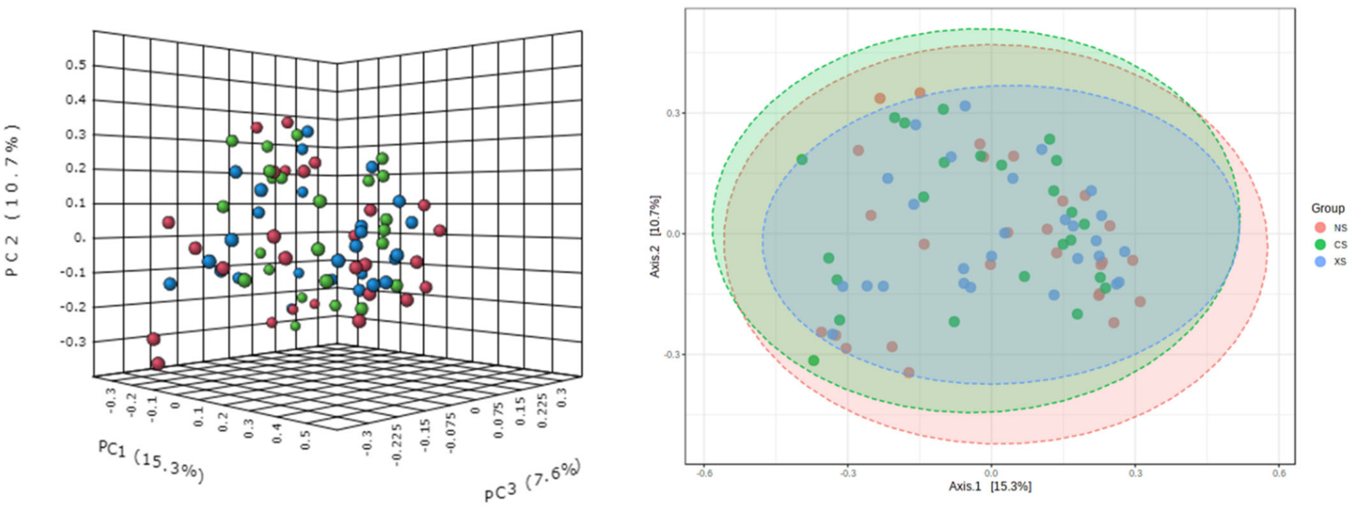

Supplemental Figure 1. Duodenal microbial alpha diversity (A) and beta diversity (B) in never-smokers (NS) (red), current smokers (CS) (green), and ex-smokers (XS) (blue). The black diamonds in A represent the mean value of each microbial index in each group.

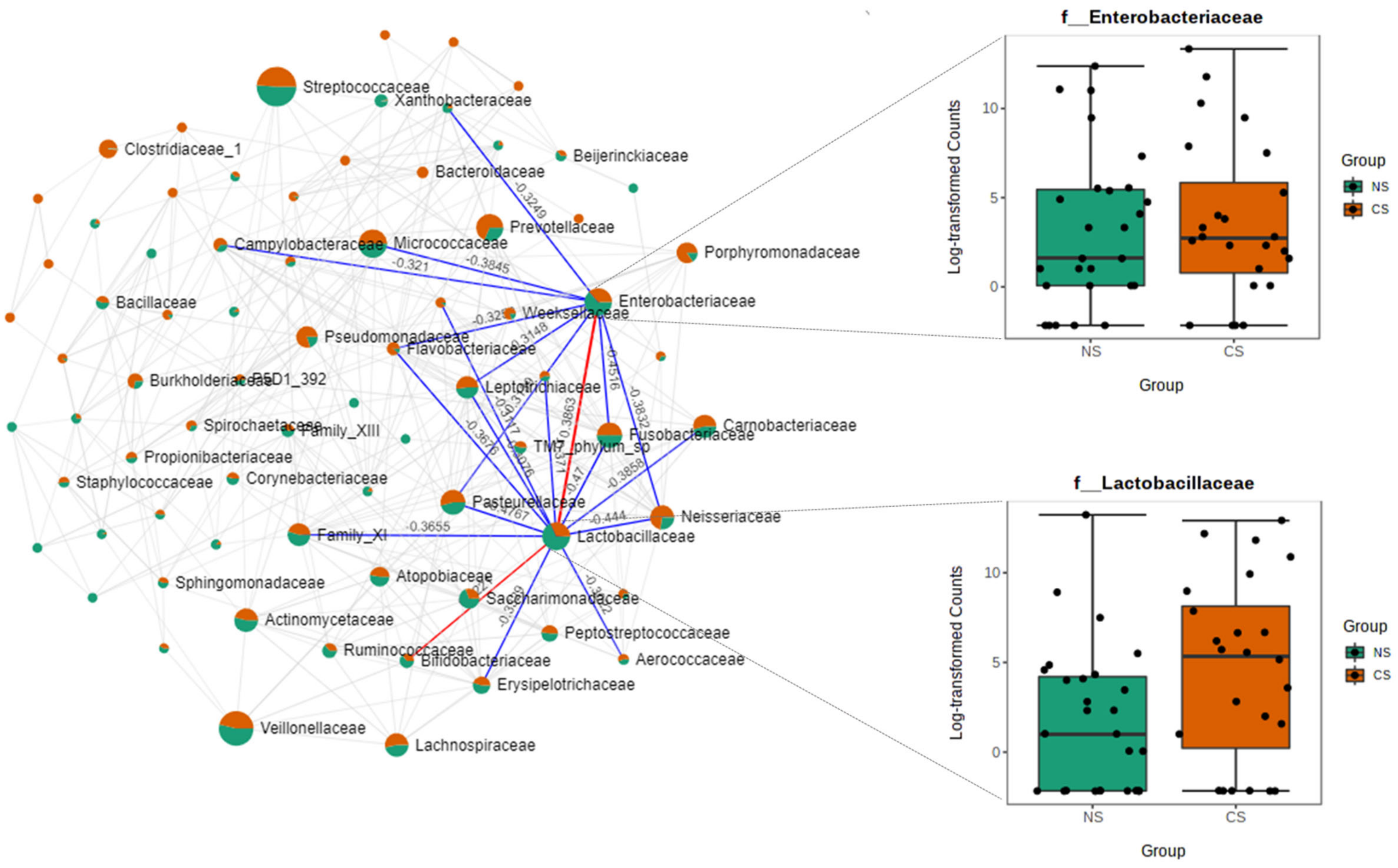

Supplemental Figure 2. Spearman correlations and relative abundances at the family level in never-smokers (orange) and current smokers (green). Blue lines represent negative correlations ($P<0.05$) and red lines represent positive associations ($P<0.05$). Log-transformed abundances of families Enterobacteriaceae and Lactobacillaceae in the duodenal luminal microbiome of never-smokers (NS) and current smokers (CS) are highlighted.

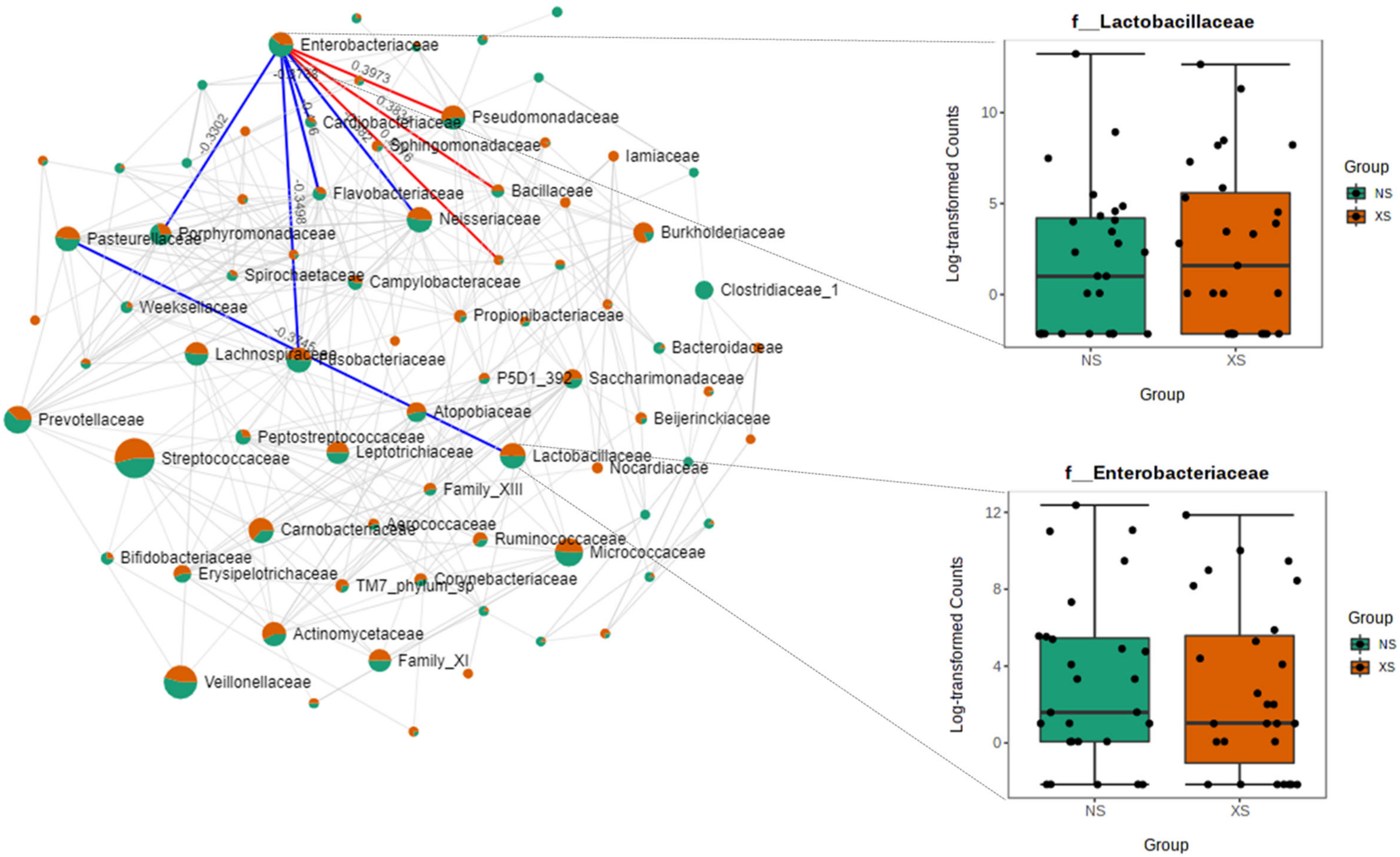

Supplemental Figure 3. Spearman correlations and relative abundances at the family level in never-smokers (green) and ex-smokers (orange). Blue lines represent negative correlations (P<0.05) and red lines represent positive associations (P<0.05). Log-transformed abundances of families Enterobacteriaceae and Lactobacillaceae in the duodenal luminal microbiome of never-smokers (NS) and ex-smokers (XS) are highlighted.

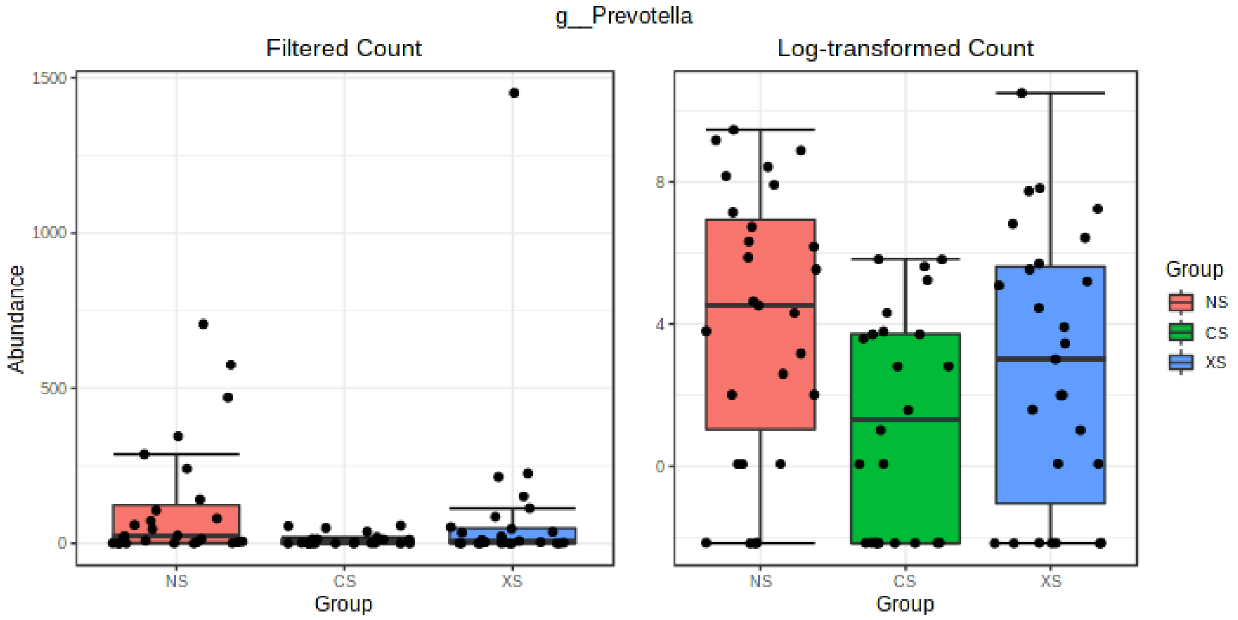

Supplemental Figure 4. Log-transformed abundance of genus *Prevotella* in the duodenal luminal microbiome of never-smokers (NS) (red), current smokers (CS) (green), and ex-smokers (XS) (blue).

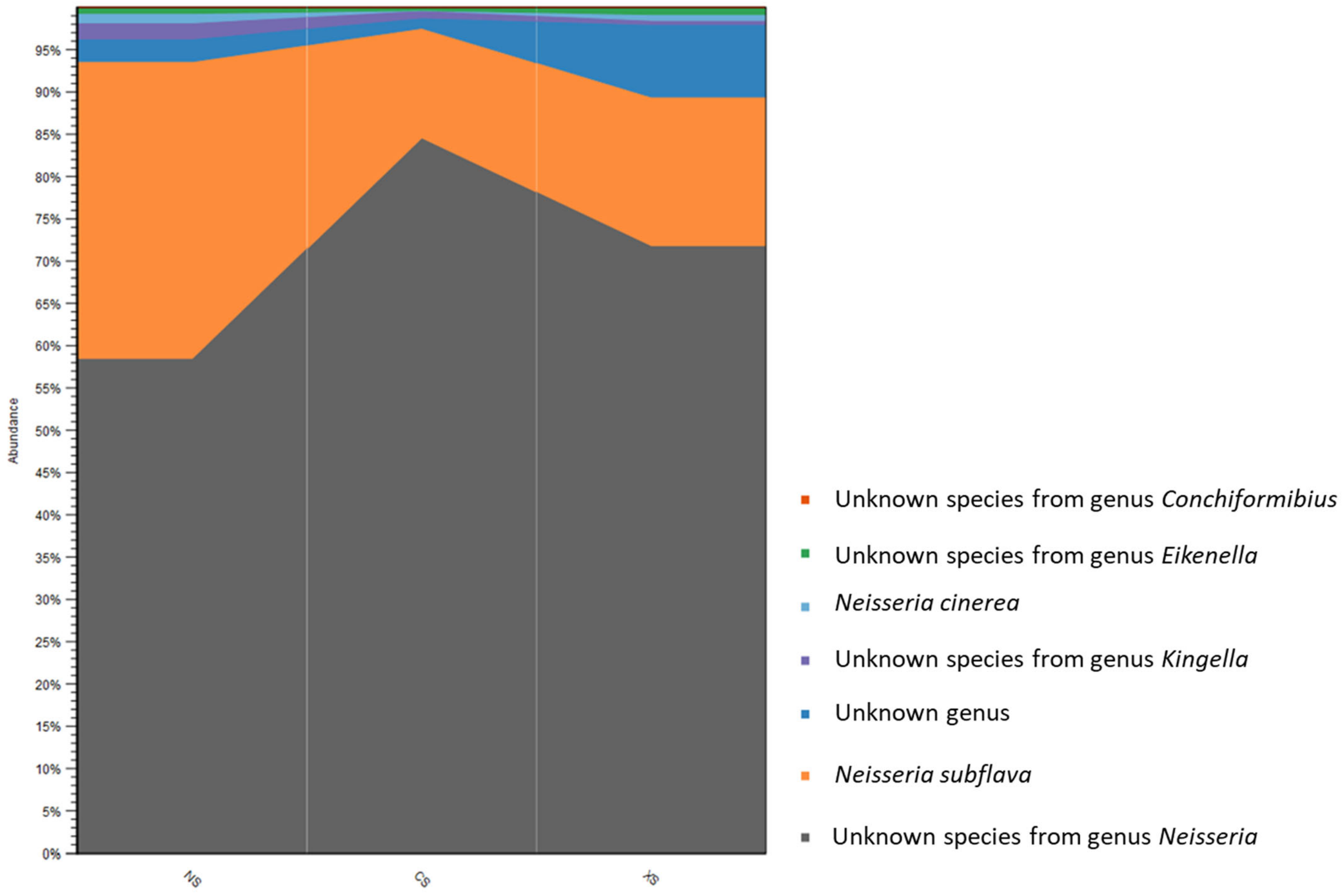

Supplemental Figure 5. Relative abundances of species within family Neisseriaceae (phylum Proteobacteria, class Betaproteobacteria, order Neisseriales) in the duodenal luminal microbiome of never-smokers (NS), current smokers (CS), and ex-smokers (XS).

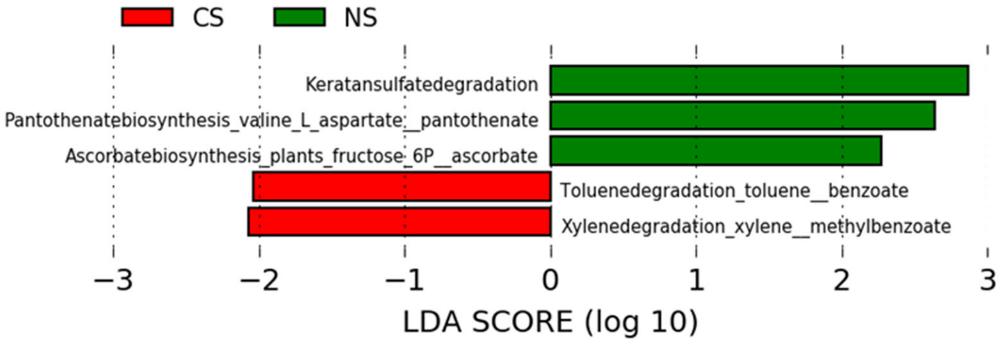

Supplemental Figure 6. Linear Discriminant Analysis (LDA) Effective Size (LEfSe) discovery analysis demonstrating the most differentially abundant PICRUSt2-predicted KEGG modules between the NS and CS groups. KEGG modules enriched in NS are indicated with a positive LDA score (green), and KEGG modules enriched in CS are indicated with a negative score (red). Only modules meeting an LDA threshold of >2 are shown.

Supplemental Table 1: Significant differences in relative abundance of small intestinal luminal microbial taxa in current smokers (CS), ex-smokers (XS) and never-smokers (NS).

**Significantly altered taxa**	**Fold change CS vs. NS**	**Fold change XS vs. CS**	**Fold change XS vs. NS**
Prevotellaceae	**-2.80**	1.41	-2.0
Neisseriaceae	**-3.41**	2.08	-1.64
Porphyromonadaceae	**-6.53**	2.20	-2.97
Enterobacteriaceae	**14.77**	**-5.87**	-3.95
Lactobacillaceae	**2.08**[1]	-5.03	-2.49
Chryseobacterium	**-4.06**	**4.44**	0.37
Lactobacillus	**4.81**	-2.17	-1.62
Unknown species, *Escherichia-Shigella*	**20.51**	**-7.68**	**-3.33**
Unknown species, *Klebsiella*	**9.90**	**-3.40**	-1.06
Lactobacillus panis	**6.40**	**-11.28**	**-4.88**
L. oris	**6.88**	**-3.08**	3.81
L. murinus	**7.28**	7.02	**10.09**
Unknown species, *Prevotella*	**-19.60**	**10.81**	**-1.81**
Neisseria subflava	**-4.17**	**2.51**	-1.66
N. cinerea	**-5.46**	**4.13**	-1.33
Unknown species, *Porphyromonas*	**-3.86**	1.83	**-2.03**
Bulleidia extructa	**4.55**	**-2.47**	**3.25**
Unknown species, *Filifactor*	**2.21**	**-5.88**	**-3.67**

Significant changes are shown in bold.

[1] This finding only reached significance when a non-parametric statistical test was used.

Supplemental Table 2: Association analysis of the top 10 PICRUSt2-predicted KEGG microbial pathways across all groups.

**Pathway**	**Hits**	**P-value**	**FDR-adj. P-value**
Glycosphingolipid biosynthesis - lacto and neolacto series	1	0.06	0.93
Flavone and flavonol biosynthesis	1	0.11	0.93
Lipopolysaccharide biosynthesis	17	0.11	0.93
Isoquinoline alkaloid biosynthesis	8	0.15	0.93
Tropane	20	1.31	1.00
Phosphonate and phosphinate metabolism	13	0.19	0.93
Bisphenol degradation	2	0.22	0.93
Linoleic acid metabolism	3	0.23	0.93
Sphingolipid metabolism	9	0.24	0.93
Polyketide sugar unit biosynthesis	5	0.25	0.93

Supplemental Table 3: Association analysis of the top 10 PICRUSt2-predicted KEGG microbial pathways between the NS and CS groups.

**Pathway**	**Hits**	**P-value**	**FDR-adj. P-value**
Flavone and flavonol biosynthesis	1	0.13	0.97
Lipopolysaccharide biosynthesis	17	0.19	0.97
Other types of O-glycan biosynthesis	1	0.20	0.97
Tropane, piperidine and pyridine alkaloid biosynthesis	9	0.22	0.97
Polyketide sugar unit biosynthesis	5	0.23	0.97
Isoquinoline alkaloid biosynthesis	8	0.26	0.97
Sphingolipid metabolism	9	0.27	0.97
Steroid hormone biosynthesis	4	0.29	0.97
Furfural degradation	6	0.29	0.97
Steroid degradation	9	0.30	0.97

Supplemental Table 4: Association analysis of the top 10 PICRUSt2-predicted KEGG microbial pathways between the CS and XS groups.

**Pathway**	**Hits**	**P-value**	**FDR-adj. P-value**
Flavone and flavonol biosynthesis	1	0.14	0.92
Secondary bile acid biosynthesis	1	0.15	0.92
Primary bile acid biosynthesis	3	0.15	0.92
Other types of O-glycan biosynthesis	1	0.15	0.92
Toluene degradation	20	0.16	0.92
Biosynthesis of type II polyketide products	3	0.18	0.92
Penicillin and cephalosporin biosynthesis	2	0.18	0.92
Fluorobenzoate degradation	10	0.18	0.92
Glycosphingolipid biosynthesis - lacto and neolacto series	1	0.19	0.92
Bisphenol degradation	2	0.20	0.92

Supplemental Table 5: Association analysis of the top 10 PICRUSt2-predicted KEGG microbial pathways between the NS and XS groups.

**Pathway**	**Hits**	**P-value**	**FDR-adj. P-value**
Lipopolysaccharide biosynthesis	17	0.09	0.65
Sphingolipid metabolism	9	0.10	0.65
Isoquinoline alkaloid biosynthesis	8	0.11	0.65
Tropane, piperidine and pyridine alkaloid biosynthesis	9	0.12	0.65
Linoleic acid metabolism	3	0.12	0.65
Bisphenol degradation	2	0.12	0.65
Glycosphingolipid biosynthesis - lacto and neolacto series	1	0.13	0.65
Ubiquinone and other terpenoid-quinone biosynthesis	34	0.13	0.65
Other glycan degradation	6	0.14	0.65
Phenylpropanoid biosynthesis	6	0.15	0.65
